# Cancer care workforce in Africa: perspectives from a global survey

**DOI:** 10.1186/s13027-019-0227-8

**Published:** 2019-05-21

**Authors:** Verna Vanderpuye, Nazik Hammad, Yehoda Martei, Wilma M. Hopman, Adam Fundytus, Richard Sullivan, Bostjan Seruga, Gilberto Lopes, Manju Sengar, Michael D. Brundage, Christopher M. Booth

**Affiliations:** 1 National Centre for Radiotherapy Oncology and Nuclear Medicine, Korle Bu Teaching Hospital, P.O Box KB369, Accra, Ghana; 2 Department of Oncology, Queen’s University, Kingston, Canada; 3 Botswana University of Pennsylvania Partnership, Gaborone, Botswana; 4 Kingston General Health Research Institute, Kingston, Canada; 5 Department of Public Health Sciences, Queen’s University, Kingston, Canada; 6 Division of Cancer Care and Epidemiology, Queen’s University Cancer Research Institute, Kingston, Canada; 7 Institute of Cancer Policy, King’s College London, & King’s Health Partners Comprehensive Cancer Centre, London, UK; 8 Division of Medical Oncology, Institute of Oncology Ljubljana, Ljubljana, Slovenia; 9 University of Miami and Sylvester Comprehensive Cancer Center, Miami, USA; 10 Department of Medical Oncology, Tata Memorial Centre, Mumbai, India

## Abstract

**Background:**

While the burden of cancer in Africa is rapidly rising, there is a lack of investment in healthcare professionals to deliver care. Here we report the results of a survey of systemic therapy workload of oncologists in Africa in comparison to oncologists in other countries.

**Methods:**

An online survey was distributed through a snowball method via national oncology societies to chemotherapy-prescribing physicians in 65 countries. The survey was distributed within Africa through a network of physicians associated with the African Organisation for Research and Training in Cancer (AORTIC). Workload was measured as the annual number of new cancer patient consults seen per oncologist. Job satisfaction was ranked on a 10-point Likert scale; scores of 9–10 were considered to represent high job satisfaction.

**Results:**

Thirty-six oncologists from 18 countries in Africa and 1079 oncologists from 47 other countries completed the survey. Compared to oncologists from other countries, African oncologists were older (median age 51 vs 44 years, *p* = 0.007), more likely to prescribe chemotherapy and radiation [61% (22/36) vs 10% (108/1079), *p* < 0.001], less likely to have completed training in their home country [50% (18/36) vs 91% (979/1079), p < 0.001], and more likely to work in the private sector [47% (17/36) vs 34% (364/1079), *p* = 0.037]. The median number of annual consults per oncologist was 325 in Africa compared to175 in other countries. The proportion of oncologists seeing > 500 consults/year was 31% (11/36) in Africa compared to 12% (129/1079) in other countries (*p* = 0.001). African oncologists were more likely than global colleagues to see all cancer sites [72% (26/26) vs 24% (261/1079), *p* < 0.001]. Oncologists in Africa were less likely than other oncologists to have high job satisfaction [17% (6/36) vs 30% (314/1079), *p* = 0.013].

**Conclusion:**

African oncologists within the AORTIC network have a substantially higher clinical workload and lower job satisfaction than oncologists elsewhere in the world. There is an urgent need for governments and health systems to improve the oncologist-to-patient ratio and develop new models of capacity building, retention and skills enhancement to strengthen the wide variety of cancer care systems across continental Africa.

## Introduction

The burden of cancer in Africa is growing with recent estimates reporting 1.06 million new cancer cases per year. This figure is expected to increase by 102% to 2.12 million by 2040 [1]. Moreover, the mortality-incidence ratio in Africa is substantially higher than high-income countries (Africa 0.66 vs Northern Europe 0.40 vs United States 0.29) [1]. This disparity in outcome is largely explained by the fact that at least 80% of patients in Africa are diagnosed with cancer at an advanced stage [2]. Other factors that likely contribute to poor cancer outcomes in Africa include limited health system infrastructure, a scarcity of specialized health personnel (including oncologists and pathologists) and patients’ inability to afford cancer treatment [3].

Given the late stages of disease at diagnosis, the vast majority of the patients in Africa are treated with palliative intent. The number of oncologists in Africa ranges from zero in countries like Lesotho, Benin, Gambia, South Sudan and Sierra Leone to single digits in Malawi, Burkina Faso, Rwanda and Togo, and up to 1500 in Egypt [4]. This translates to a very high case-load per physician. Moreover, with the scarcity of medical oncologists (MOs) in Africa and other low-middle income regions, a large proportion of cancer patients may not even have the opportunity to see a medical oncologist in consultation. These patients therefore do not have access to early interventions to reduce suffering and improve outcomes.

A recent review included data from 21 countries in Africa, and showed a high burden of incident cases per oncologist while also highlighting the large numbers of oncologist in Africa who practice both radiation and medical oncology (i.e. clinical oncologists) further compounding the workload [4]. Four studies have explored medical oncology workload in high-income countries (HICs) [5–8]. In these studies from New Zealand, The United States, Australia, and Canada the MO case-load was 175–280 new patients/year. We have recently reported the results of a global analysis of MO workload in which we observed striking difference in case volumes between HICs and low-middle income countries (LMICs) [9].

To address gaps in knowledge, we report a sub-set analysis of a global study in which we describe: 1) the clinical workload of African oncologists compared to those of other countries; 2) available infrastructure and supports; and 3) delivery of clinical care in the African context. Data from this study will inform cancer policy and human resource planning in Africa.

## Methods

### Study population

We have recently reported the results of our global study of medical oncology workload [9]. The study population for the global study included any practicing physician who delivers chemotherapy; trainees were not eligible. The web-based survey was distributed using a modified snowball methodology to oncologists in 54 countries and 2 regional networks (Caribbean and Africa). Contact was preferentially directed to established national associations of medical/clinical oncologists; if this was not possible we approached one personal contact per country to invite participation and distribute the survey via an informal national network. The survey was distributed to oncologists in Africa through the network of the African Organization for Research and Training in Cancer (AORTIC). In Africa, the respondents were mostly clinical oncologists trained in both medical oncology and radiation oncology. The global study included 1115 participants from 65 countries. Thirty-six oncologists from 18 countries in Africa participated in the study; they form the primary cohort described in the current analysis and are compared to 1079 participants from 47 other countries. This study was approved by the Research Ethics Board of Queen’s University.

### Survey design and distribution

An on-line electronic survey questionnaire was developed via Fluid Surveys to capture the following information: participant demographics, clinical practice setting, clinical workload, and barriers to patient care. The survey was designed with multidisciplinary input of the study investigators. A complete survey was then piloted and subsequently revised based on feedback from 10 additional oncologists. The final survey included 51 questions and took 10–15 min to complete.

Distribution of the global survey utilized two primary methods. The senior investigator (CMB) contacted individuals and regional oncology associations in order to create a broad distribution network. Whether the national contact was an association or an individual, they were provided with an electronic link to the survey to distribute to their national membership/network. These links were unique to each nation, but not individualized. The distributing partners were asked to provide the research team with the number of survey recipients to ascertain the national response rate. The survey was distributed in November 2016. A second reminder email was sent via all national contacts in January 2017.

### Statistical analysis

Countries participating in the global study were classified into low or low-middle (LMIC), upper-middle (UMIC) and high-income countries (HIC) based on World Bank criteria [8].The results of respondents who identified African countries of practice were extracted and analysed as a single group. These results were then compared against the results from the other 47 countries that participated in the global study. The primary objective was to describe the workload of African oncologists compared to oncologists practicing in other countries. Workload was defined as the annual number of new cancer patient consults seen per oncologist. All data were initially collected in Fluid Surveys and subsequently exported to IBM SPSS (version 24.0 for Windows, Armonk, New York, 2016). Data consisted of categorical, ordinal and continuous formats, occasionally collected as ranges (e.g. < 50, 51–100, 101–150, etc.). Pearson chi-square tests or Fisher’s Exact tests were used to test for differences in proportions for categorical variables, and the Mann-Whitney U was used to compare ordinal and continuous data between African and other countries. *P*-values of < 0.05 were deemed statistically significant. No adjustments were made for multiple comparisons.

## Results

### Survey distribution and response

Fifty-four countries and two regional networks (Africa and Caribbean) were invited to participate in this study; 42 (75%) agreed to participate. Overall, 1115 respondents from 65 different countries participated in this study. Survey response rates were available for 40% (17/42) of all countries/regional networks and ranged from 3% in Singapore and Portugal to 76% in Slovenia (Appendix 2). Thirty-six respondents practiced in 18 African countries, including respondents from Algeria (1), Angola (1), Botswana (1), Burkina Faso (1), Central African Republic (1), Egypt (3), Ghana (5), Ivory Coast (1), Kenya (1), Morocco (1), Mozambique (2), Namibia (1), Nigeria (7), South Africa (3), Sudan (3), Tanzania (2), Uganda (1) and Zambia (1). The response rate in Africa was not known since it is not known how many individuals within the regional network received the survey but did not complete it.

### Characteristics of study participants

Compared to other countries, oncologists in Africa were older (median age 51 vs 44 years, *p* = 007) and more likely to be male [72% (26/36) vs 58% (621/1079), *p* = 0.085] (Table 1). Oncologists in Africa worked in lower income countries [81% (29/36) LMIC and 19% (7/36) UMIC] compared to other respondents [11% (118/1079) LMIC, 17% (179/1079) UMIC and 73% (782/1079) HIC]. Practitioners in Africa were more likely to be Clinical Oncologists [56% (20/36) vs 9% (92/1079), p = < 0.001] and less likely to be Medical Oncologists [22% (8/36) vs 83% (890/1079, *p* < 0.001] compared to respondents from elsewhere. African oncologists were also more likely to prescribe chemotherapy and radiotherapy [61% (22/36) vs 10% (108/1079), *p* < 0.001]. Respondents in Africa were less likely to have completed training in their home country [50% (18/36) vs 91% (979/1079), *p* < 0.001].

**Table 1:** Demographics and clinical practice setting of respondents from Africa and other countries to a global medical oncology workload survey (*n* = 1115)

	Africa *N* = 36	Other Countries *N* = 1079	*P*-Value
	N (%)		
Demographics			
Sex			
Male	26 (72)	621 (58)	0.085
Female	10 (28)	453 (42)	
Age (median)	51	44	0.007
World bank country income level			
High-income	0 (0)	782 (73)	< 0.001
Upper-middle income	7 (19)	179 (17)	
Low- and low-middle income	29 (81)	118 (11)	
Years in practice (median)	13	10	0.209
Specialty			
Medical Oncologist	8 (22)	890 (83)	< 0.001
Clinical Oncologist	20 (56)	92 (9)	
Pediatric Oncologist	1 (3)	10 (1)	
Hematologist	5 (14)	44 (4)	
Other^	2 (6)	43 (5)	
Years of post-graduate training (median)	6	6	0.147
Completed training in Home country			
Yes	18 (50)	979 (91)	< 0.001
No	18 (50)	91 (8)	
No response	1 (2)	9 (1)	
Clinical Practice Setting			
System			
Public	19 (53)	714 (66)	0.037
Private	4 (11)	160 (15)	
Both	13 (36)	204 (19)	
Setting			
Hospital In-Patient	30 (52)	552 (76)	< 0.001
Hospital Outpatient	54 (93)	649 (90)	0.502
Other Outpatient	7 (12)	57 (8)	0.262
Hospital type			
General Hospital	17 (47)	707 (66)	0.048
Cancer Hospital	19 (53)	360 (33)	
Not in hospital setting	0	3 (0)	
Radiotherapy on Site			
Yes	26 (72)	894 (83)	0.149
No	10 (28)	173 (16)	
Palliative Care on Site			
Yes	27 (75)	896 (83)	0.289
No	9 (25)	171 (16)	
Chemotherapy Pharmacist on Site			
Yes	26 (72)	895 (83)	0.137
No	10 (28)	171 (16)	
Training Program in Center			
Yes	24 (67)	799 (74)	0.322
No	12 (33)	280 (26)	
Supervise trainees			
Yes	30 (83)	913 (85)	0.834
No	6 (17)	166 (15)	
EMR			
Yes	8 (22)	894 (83)	< 0.001
No	28 (78)	178 (17)	
Clinic notes ^ῲ^			
Dictated	4 (11)	359 (33)	0.005
Hand-Written	34 (94)	363 (34)	< 0.001
Typed	6 (17)	630 (58)	< 0.001
Service Extenders ^ῲ^			
Nurse	30 (83)	788 (73)	0.169
Nurse practitioner	18 (50)	547 (51)	0.935
Medical Students	13 (36)	307 (29)	0.318
Residents	27 (75)	661 (61)	0.095
Other Physicians	12 (33)	291 (27)	0.398

Numbers do not always add to 100% due to rounding or small amounts of missing data. Responses are missing for sex (*n* = 5), age (n = 5), clinical practice setting (system) (n = 1), radiotherapy on site (*n* = 12), palliative care on site (*n* = 12), chemotherapy pharmacist on site (*n* = 13), access to Electronic Medical record (EMR) (*n* = 7)

^ῲ^ Indicates applicants could choose multiple responses to same question

^Other providers included internal medicine (*n* = 3) and gynecology (*n* = 3)

### Clinical practice setting

African oncologists were more likely than other respondents to work in the private sector [47% (17/36) vs 34% (364/1079), *p* = 0.037] and have fewer systemic therapy colleagues within their unit. Radiotherapy, palliative care, and chemotherapy pharmacist support were available on site in 72% (26/36), 75% (27/36), and 72% (26/36) of African units who responded; this did not differ substantially from global rates. However, African cancer units were significantly less likely to have electronic medical records [22% (8/36) vs 83% (894/1076), *p* < 0.001] than elsewhere.

### Delivery of clinical care

African oncologists reported working a median of 45 h per week with four weeks annual leave for vacation and 1.5 weeks annual leave for conference; these are comparable to metrics from other countries (Table 2). African oncologists were on-call 7 days per month compared to 3 days/month in other countries (*p* = 0.002). The proportion of time that African oncologists spend on clinical duties (mean 62%), research (mean 14%), teaching (mean 13%), and administration (13%) is consistent with oncologists in other countries. African oncologists were more likely than global colleagues to see patients with all cancer sites [72% (26/36) vs 24% (261/1079), *p* < 0.001].

**Table 2:** Delivery of clinical care and clinical volumes* reported by respondents from Africa and other countries to a global medical oncology workload survey (*n* = 1115)

	Africa N = 36	Other Countries *N* = 1079	*P*-Value
Delivery of clinical CARE			
Work Week			
No. Days Worked/Week (median)	5	5	0.882
No. Hours Worked/Week (median)	41–50	41–50	0.642
Leave			
No. Annual Weeks of Vacation (median)	4	4	0.195
No. Annual Weeks Conference Leave (median)	1.5	2	0.433
On-Call Duties**^**			
No. Days On-call/month (median)	6.5	3	0.002
Respondents On-Call Every Night (n (%))	10 (35)	193 (26)	0.284
Allocation of Duties			
% Time on Clinical Duties (mean)	62	63	0.756
% Time on Research (mean)	14	14	0.825
% Time on Teaching (mean)	13	9	< 0.001
% Time on Administration (mean)	13	14	0.650
Clinical Volumes			
No. Annual New Consults (median)	325	175	0.001
**≤** 100 *N* (%)	6 (17)	284 (26)	
101–200	7 (19)	351 (33)	
201–300	4 (11)	189 (18)	
301–400	5 (14)	62 (6)	
401–500	3 (8)	52 (5)	
> 500	11 (31)	129 (12)	
No. Patients Seen Per Clinic Day (median)	25	25	0.035
< 10 *N* (%)	2 (6)	107 (10)	
10–20	12 (33)	423 (39)	
21–30	8 (22)	322 (30)	
31–40	7 (19)	120 (11)	
> 40	7 (19)	102 (9)	

Small amounts of data were missing for number of annual new consults (*n* = 12) and number of patients seen per clinic day (*n* = 5). On call duties had a large amount of missing information, with only 29 respondents from Africa and 754 respondents from other countries replying to this item

*Per full day of out-patient clinic

### Clinical volumes

The median number of new patient consults per year among African oncologists was 325 compared to 175 for oncologists practicing in other countries (*p* = 0.001) (Table 2). The proportion of oncologists seeing > 500 consults/year was 31% (11/36) in African countries compared to 12% (129/1079) in other countries. The median number of patients seen in a full day of clinic in surveyed African countries was 25; 19% (7/36) saw > 40 patients per day compared to 9% (102/1079) in other countries (*p* = 0.035). Eighty-three percent (30/36) of African oncologists attended regular weekly multidisciplinary case conferences.

### Satisfaction, barriers and challenges

The median job satisfaction score on a 10-point Likert scale (higher scores represent higher satisfaction) was 7 in Africa and 8 in other countries. Oncologists in Africa were less likely than other oncologists to have high job satisfaction (Likert score 9 or 10); 17% (6/36) vs 30% (314/1079), *p* = 0.013.The most common barriers to clinical care reported by oncologists from Africa included patients being unable to pay for treatment (50%, 18/36), high clinical volumes (44%, 16/36), limited access to new treatments (39%, 14/36), limited access to radiotherapy (33%, 12/36), and limited access to chemotherapy (28%, 10/36) (Table 3).

**Table 3:** Top five reported barriers to patient care as reported by African respondents to a global medical oncology workload study compared to respondents from other countries (*n* = 1115)

Africa (*n* = 36)	*s* (%)	Other countries (*n* = 1079)	*N* (%)
Patients unable to pay	18 (50)	High clinical volumes	639 (59)
High clinical volumes	16 (44)	Insufficient time for reading	421 (39)
Access to new treatments	14 (39)	Access to new treatments	357 (33)
Unavailability/limited access to radiotherapy	12 (33)	Shortage of oncologists	315 (29)
Unavailability/limited access to chemotherapy	10 (28)	Shortage of nurses	257 (24)

## Discussion

In this study we describe the clinical workload, infrastructure and delivery of care among African clinical oncologists. For comparative purposes we also present data from 47 other (predominantly high-income) countries. Several important findings emerged. First, the clinical workload is substantially higher among African oncologists compared to oncologists in other countries. Second, African oncologists are substantially older than oncologists in other countries which suggests that without new models of care and an increase in capacity, clinical workload volumes might worsen in the coming years. Third, job satisfaction among African oncologists is lower than oncologists in other countries. This raises the concern of physician burn-out which could further exacerbate human resource challenges. Finally, the most important barriers to oncologic clinical care reported by African oncologists are the inability of patients to pay for care, high clinical volumes, limited access to new therapies, standard chemotherapy and radiotherapy.

We have recently completed the first global analysis of medical oncology workload and available infrastructure [9]. In our global analysis we found striking differences in workload and delivery of care among countries in different World Bank income categories. Annual case volume in LMICs (median consults of 425, 40% respondents seeing > 500 consults) was substantially higher than UMICs (median consults of 175, 14% seeing > 500 consults) and HICs (median consults of 175, 7% > 500 consults) (*p* < 0.001). The results presented in this study confirm these disparities in oncology workload, given that most of the African respondents are from LMICs and the other respondents in our comparative group are predominantly from HICs.

Our results are consistent with previous reports highlighting the scarcity of cancer treating physicians in Africa [10], and are also similar to findings from a recent study published by Mathew [4] which evaluated the global clinical oncology workforce by estimating the oncologist-to-cancer burden ratio in 93 countries, including 21 from countries in Africa. The new consult volumes per oncologist in Mathew’s report are substantially higher than reported in our analysis. Whereas we report a median of 325 new consults per oncologist per year, Mathew reports several countries in Africa with workloads greater than 1000 new cancer cases per oncologist per year. While Mathew’s analysis are based on the International Agency for Research on Cancer estimates, our results are based on survey responses which reflect the actual clinical burden, as opposed to projected estimates of new cancer diagnoses and our results may therefore be more representative of the clinical oncology workforce burden. A significant proportion of patients diagnosed in Africa do not come into contact with the cancer health system. Therefore not all of the estimated incident cases are seen by an oncologist. However we do anticipate that with increase in awareness the clinical volume and cancer workload will increase substantially.

Our results also highlight not only the magnitude of workload but also the complexity of cancer care delivery in Africa; most cancer physicians prescribe chemotherapy and radiotherapy and treat patients with all cancer sites. In Africa this model may be driven by limited resources, however, the joint practice and accreditation of clinical oncology to cover both chemotherapy and radiotherapy is standard for many countries including the United Kingdom. Thus this may also reflect clinical practice and training based on Commonwealth and other structures. A 2014 survey by the European Society for Radiotherapy and Oncology revealed that 75% of radiation oncologists also administered systemic cancer therapies [11]. The United Kingdom still continues to train dual-prescribing clinical oncologists alongside medical oncologists. However, realizing the evolving complexities of systemic cancer therapy and radiation therapy in the modern era, the European Union issued a directive in 2011, recognizing medical oncology as a separate entity from radiation oncology [12].

Another important finding from our analysis is job satisfaction is lower among African oncologists compared to other countries. This may relate to high clinical workload, the complexity of delivering care in low resource settings, and poor compensation. These trends may precipitate oncology burnout in Africa, contribute to brain drain among African oncologists [13], and lead to fewer African physicians willing to specialize in oncology [14].

With the expected future increase in cancer incidence and a disproportionate burden of disease in LMICs, this survey highlights an important healthcare personnel gap in scaling up cancer control programs in Africa. The combination of increased workload, complexity of cancer care, lower job satisfaction rates and projected increases in cancer incidence calls for continued growth in the oncology workforce in addition to developing new models of care that make use of service extenders. The recent 2017 World Health Organization cancer resolution to improve access to quality cancer care, cannot be realized in LMICs without pragmatic strategies to increase the cancer care delivery workforce [15, 16]. Realistically the stagnant growth in the clinical oncology workforce will not be sufficient to manage the projected increase in the burden of disease in the region. Therefore in these resource-limited settings, predominantly in sub-Saharan Africa, mechanisms for using service extenders and task shifting is paramount, but should be done within an implementation science framework to ensure these adaptive models do not compromise delivery of high quality care. Countries like Rwanda have experimented with task shifting and re-training of general practitioners with reported success on a small scale [17].

While a significant proportion of African respondents reported training outside of their country compared to respondents from other countries, 67% reported having training programs in their center. For many years the International Atomic Energy Agency, supported the training of clinical oncologists from many LMICs and UMICs (including physicians from African centers with existing RT facilities) to be trained in HICs [18]. At the time, this model was thought to be a cost-effective approach to improving the cancer workforce. However, this meant that centers without planned radiotherapy facilities were without a clinical oncologist to manage solid tumors. Our data may therefore reflect a scaling up of oncology training centers across Africa. This shift in training African oncologists on the continent has been accompanied by significant reductions in the cost of training and is expected to curb the rates of brain drain [19]. Currently there is no database of oncology training centers on the continent, however the survey results and our clinical experience in Africa, are consistent with an increase in new and improved local training opportunities in medical oncology and the formation of regional training centers of excellence across the continent [20, 21].

Cancer care is complex and requires a multidisciplinary approach. The barriers highlighted here suggest that in addition to resource allocation and healthcare planning to expand the cancer care workforce, there should be a parallel focus to improve access to high-quality radiotherapy, surgery, and palliative care. Special focus is needed to identify funding for cancer medicines that offer substantial and clinically meaningful benefit. Moreover, availability of diagnostic platform systems such as radiology and pathology are severely limited across Africa [22] and require increased capacity to meet the population needs. Recent global data also describe the association between high clinical volumes and low job satisfaction [23]. Further work is required to understand the risk of burn-out among cancer care providers in the African context.

Our study results should be considered in light of important methodologic limitations. Due to the potential for selection bias, it is possible that our results are not generalizable to all African countries; this is particularly true given the small sample size. Our survey was delivered within the AORTIC network and therefore we are unable to assess a denominator to estimate the response rate to our survey. There is the possibility of a selection bias as countries with allocated resources for oncology may be disproportionately represented in AORTIC. This is however likely to result in a bias of our results towards the null, and the oncology workforce burden in Africa might be much larger than our survey results show. Finally, the workload metric (new consults per year) is a crude marker of clinical workload as the work involved across cancer sub-types and patient populations can vary substantially.

In summary, this study offers some insight into oncology workload and delivery of cancer care in Africa. Our data identify a significant gap in healthcare personnel. AORTIC is currently mapping out regional oncology training facilities, centers of excellence and oncology workforce in Africa with the aim of establishing an accurate database for future cancer care planning. Future work is needed to explore innovative models of care in order to mitigate the clinical workload and ensure high-quality care for patients in Africa.
